# Mental Health and Suicide Attempts in Adolescents: A Systematic Review

**DOI:** 10.3390/healthcare13091039

**Published:** 2025-05-01

**Authors:** Virginia Morcillo, Maria Ferrer-Ribot, Bartomeu Mut-Amengual, Sara Bagur, Maria Rosa Rosselló

**Affiliations:** Department of Applied Pedagogy and Educational Psychology, University of the Balearic Islands, 07122 Palma, Spain; maria.ferrer-ribot@uib.es (M.F.-R.); tomeu.mut@uib.es (B.M.-A.); sara.bagur@uib.cat (S.B.); mrosa.rossello@uib.es (M.R.R.)

**Keywords:** suicide, systematic review, adolescence, prevention, risk factors

## Abstract

Suicide has become one of the leading causes of mortality among the adolescent population. This worrisome fact demands an exhaustive analysis of this social phenomenon’s reality. Objective: This paper aims to carry out a systematic review of the existing scientific literature in recent years on suicide among minors and adolescents. Method: To achieve this purpose, central databases, WoS, Scopus, and Dialnet, were analyzed, obtaining 34 articles. Results: The results are structured into three main categories. (1) Risk factors: psychological and mental health factors, family and context factors, and, finally, school factors. (2) Prevention: School as prevention, emotional education programs, and tools to deal with this situation. And, finally, the third block, (3) family experiences in the face of suicidal behavior. Conclusions: The studies reviewed highlight the enormous complexity of this problem and show a worrying situation regarding suicide, considering multifactorial aspects such as depression, psychosocial issues, and mood disorders, as well as causes related to the school environment, such as bullying and lack of connection with school. In addition, they denote the importance of family factors in this aspect. Finally, the results highlight the importance of prevention and early detection, underlining the need to develop concrete actions to alleviate this growing situation among adolescents.

## 1. Key Practitioner Message

### 1.1. What Is Known?

Suicide is a significant concern among adolescents: Suicide has emerged as one of the leading causes of death among adolescents globally. Scientific evidence indicates that the COVID-19 pandemic has significantly intensified this trend. The pandemic has not only exacerbated pre-existing risk factors, such as social isolation, emotional distress, family economic hardship, and limited access to mental health services, but has also introduced new psychosocial stressors, including the prolonged closure of educational centers, increased digital exposure, and the weakening of social support networks. These combined factors have contributed to a rise in suicidal ideation, suicide attempts, and self-harming behaviors among adolescents worldwide, underscoring the urgent need to reassess traditional prevention and intervention strategies in the post-pandemic context. Several factors, including psychological, family, and school-related issues, continue to contribute to the high risk of suicidal behavior in this age group.

Multifactorial nature of suicide risk: the phenomenon of suicide is complex and influenced by multiple interrelated factors, such as depression, bullying, family neglect, and lack of peer support.

### 1.2. What Is New?

A comprehensive systematic review of risk factors and interventions: this study adds to the existing body of literature by presenting a systematic review of recent findings on adolescent suicide, focusing on updated data and the influence of the COVID-19 pandemic on suicide rates.

Identification of family and school factors: the study highlights the critical role of family dynamics and school environments, with an emphasis on how emotional neglect and bullying exacerbate suicide risks in adolescents.

### 1.3. What Is Significant for Clinical Practice?

Importance of early detection and prevention: Clinical professionals should prioritize the early identification of suicidal behaviors, mainly through school-centered interventions and family support systems. Addressing psychological distress and promoting emotional well-being in adolescents is a top priority. In this regard, it is essential to consider the impact of the COVID-19 pandemic as a significant factor in clinical practice. The pandemic has transformed the psychosocial environment of adolescents, leading to increased rates of social isolation, emotional distress, and academic disengagement, all of which are well-documented risk factors for suicidal behavior. Therefore, clinical assessments must incorporate pandemic-related stressors as part of the evaluation of suicide risk. Likewise, prevention programs and therapeutic interventions should be adapted to address the vulnerabilities that the pandemic has exacerbated or generated, emphasizing trauma-informed care, resilience-building strategies, and strengthening social support networks. Ignoring the pandemic’s impact would risk underestimating the current mental health needs of adolescents and could compromise the quality of clinical responses in the post-pandemic context. Thus, adapting prevention and intervention strategies to the specific psychosocial challenges faced by adolescents becomes urgent.

Multidisciplinary approach in interventions: Given that adolescent suicide is often the result of multifactorial causes, professionals must consider the various interrelated factors that may influence attitudes and behaviors related to suicide in adolescents. High-quality intervention requires a holistic and coordinated approach that addresses not only individual mental health needs but also the broader social and environmental context that shapes adolescent well-being.

## 2. Introduction

The persistence and extent of the phenomenon of suicide is a widespread issue in all societies. It is a complex and multi-causal phenomenon that requires urgent attention [1]. According to data provided by the World Health Organization [2], it is estimated that around 703,000 people die by suicide annually, with a considerable number of attempts. Suicide can manifest itself at any stage of life, being the fourth leading cause of death in the 15–29 age group globally in 2019. The recent context of the COVID-19 pandemic is not unrelated to this unusual increase in admissions for suicide attempts in young people and adolescents, going from four attempts per week to more than twenty [3]. 

The United Nations’ and the WHO’s Comprehensive Plan of Action on Mental Health 2013–2030 has set a goal of reducing the global suicide mortality rate by one-third by 2030, setting this target as a key mental health indicator within the Sustainable Development Goals (SDGs). This initiative is reflected in the Implementation Guide for Suicide Prevention in Countries, designed by the Pan American Health Organization [4]. 

Given the data provided by these international organizations, it is necessary to consolidate, as a priority on the political agenda, the guarantee of access to quality mental health services for all minors and adolescents in any nation. Although suicides become the most dramatic manifestation of mental health problems, focusing solely on these numbers leaves numerous unrecorded ideas and attempts, as well as undiagnosed mental and behavioral disorders, unaddressed. These elements generate considerable emotional suffering in minors and adolescents, as well as in their families [5]. 

In this context, this study aims to carry out a systematic review of the updated scientific literature, to analyze and evidence risk factors such as preventive interventions associated with suicidal behavior in adolescents and minors at the international level, and to provide a comprehensive view of the state of knowledge in this area of mental health.

## 3. Method

The present research was based on an original systematic literature review following the PRISMA protocol [6]. The process followed three phases: (1) document search and evaluation, (2) selection and filtering, and (3) document analysis.

### 3.1. Search

A study search was conducted in three electronic databases (WoS, Scopus, and Dialnet Plus) during May 2023 by S.B. and M.F.-R. The key concepts were (1) suicide, (2) childhood, and (3) education. Table 1 shows the search equations and initial results.

### 3.2. Evaluation of Initial Results

With the initial results, S.B. and M.R.R. conducted a bibliometric analysis with VOSViewer (Centre for Science and Technology Studies, Leiden University, The Netherlands. Version: 1.6.20) to evaluate the search’s effectiveness. To this end, bibliometric maps of keyword co-occurrence are presented. The minimum number of occurrences wasset with *f*(KW) ≥ 5. 

**Figure 1:**
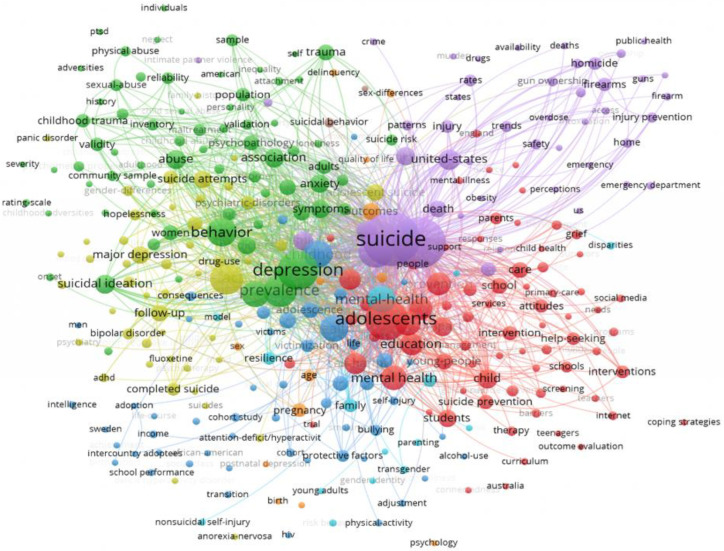
WoS cluster.

In the case of WoS (Figure 1), 8 clusters of 327 items were identified. The total weight of the 10,443 interconnections was 22,626. About the study objective, Cluster 5 (purple) interconnected “Suicide” (314 links, 2415 total strength, 367 occurrences) and “Child*” (297 links, 2101 total strength, 318 occurrences), with these being the two KW with the highest total weight compared to the other KW. “Educat*” was in Cluster 1 (red) (148 links, 372 strength, 60 occurrences) with a lower total weight. However, 92.3% of their relationships were with Cluster 5.

**Figure 2:**
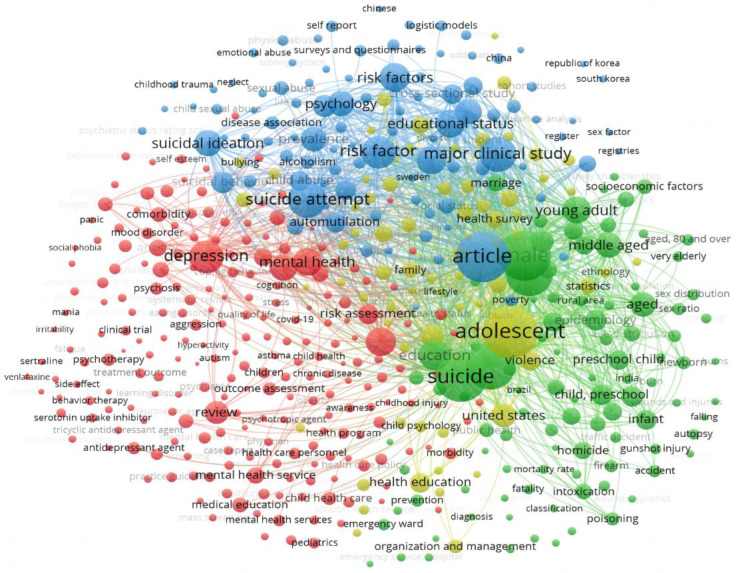
Scopus cluster.

In the case of Scopus (Figure 2), due to the high number of KW, the results were reduced to the first 500 KW with the highest reach force. Four clusters consisting of 497 items were identified. The total weight of the 579,763 interconnections was 87,145. Concerning the study objective, Cluster 2 (green) interconnected “Suicide” (496 links, 27,423 total strength, 1364 occurrences), “Child*” (496 links, 27,032 total strength, 1278 occurrences), and “Educat*” (496 links, 10,961 strength, 540 occurrences), with these being the first three KW with the highest total weight compared to the other KW, even though "Adolescent" found in Cluster 4 had a higher total weight.

### 3.3. Eligibility Criteria

The selection criteria included research articles published between 2021 and 2023, a period chosen to capture the most recent developments in adolescent suicide research, particularly considering the impact of the COVID-19 pandemic on adolescent mental health. Studies written in either English or Spanish were considered for inclusion. The incorporation of Spanish-language publications was based on the research team’s expertise regarding adolescent suicide in Spain and other Spanish-speaking countries, ensuring the cultural and epidemiological relevance of the review. This decision was particularly pertinent given that, according to data from the Spanish National Statistics Institute (INE), suicide has become the leading cause of external death among adolescents in Spain, with a significant increase following the pandemic. Nevertheless, the final selection of studies predominantly included publications in English. This outcome reflects both the greater volume and the broader international dissemination of research on adolescent suicide in English-language journals, as well as the application of rigorous quality criteria during the screening and selection process.

### 3.4. Study Screening and Selection

**Figure 3:**
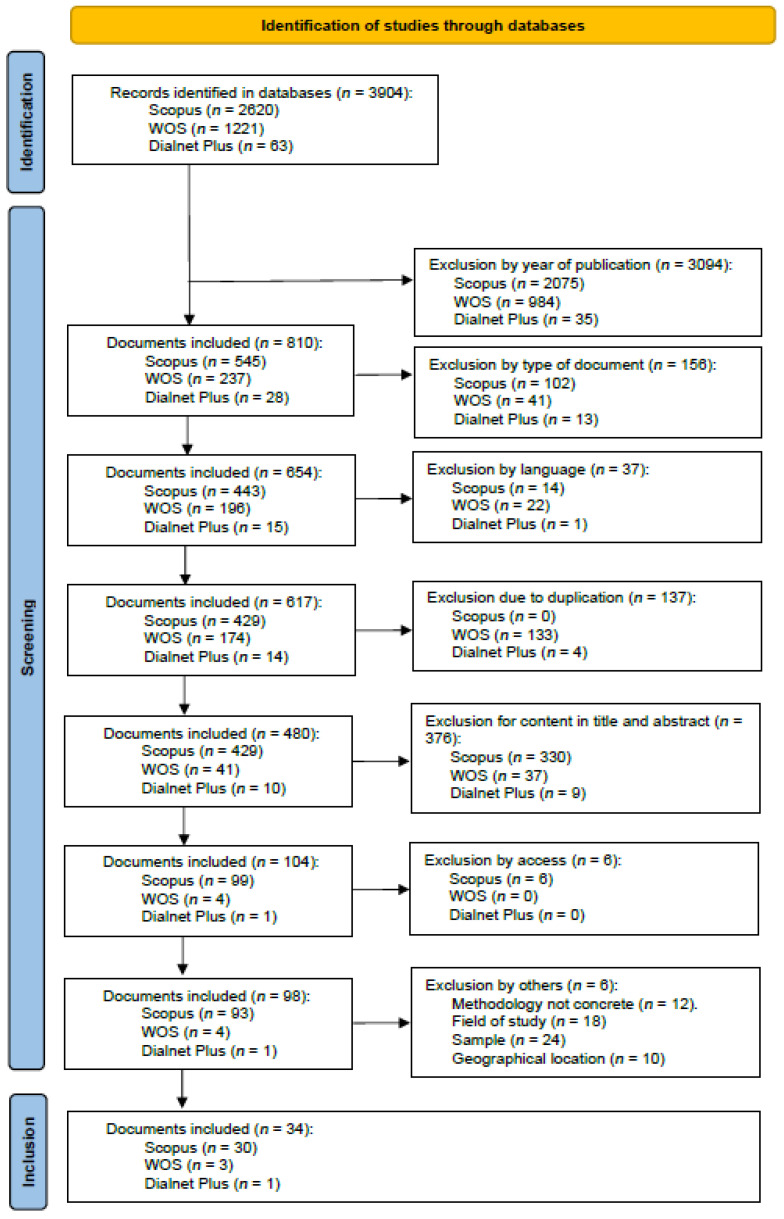
Flowchart.

We selected studies that addressed suicide prevention in school performances, early identification of suicidal behavior, risk factors for suicide at early ages, and characteristics of suicidal behavior (Figure 3). Three authors (B.M.-A., V.M., and M.R.R.) carried out an independent review exercise on titles and abstracts according to the criteria for document integration. It was put together, producing a degree of agreement of 91%. 

Source: as shown in Figure 3, the flow diagram outlines the selection process for the included studies.

### 3.5. Data Extraction and Quality Assessment

Two authors (B.M.-A. and V.M.) prepared a table with the characteristics of the final documents (Table 2) (*N* = 34). Following the PRISMA, S.B. and M.R. independently evaluated document quality aspects. The results were pooled, revealing an 87% match. 

## 4. Results

### 4.1. Risk Factors

A review of various studies reveals that the risk factors associated with suicidal behavior do not originate from a single factor but are the result of the interaction and interrelationship of several elements that contribute to the risk of suicide (for a brief overview, see Appendix A, Table A1).

#### 4.1.1. Psychological and Mental Health Factors

Mood disorders, depression, and psychosocial and attention problems have been identified as key determinants of mental health in children and adolescents [12,14,21]. The evaluation of the HiTOP model in this population has identified five primary psychopathology factors: fear, distress, externalization, thought disorder, and traumatic stress, establishing specific associations between trauma types and various clinical dimensions. Additionally, it has been demonstrated that health perception in adolescents with suicidal ideation progressively declines with age, while depressive conditions exacerbate [25,28]. Individual factors such as callousness and negative affect have been linked to suicidal ideation [23]. In contrast, higher levels of effortful control appear to mitigate this risk, compared to high negative emotionality, which significantly increases it [22]. Furthermore, interpersonal microaggressions have been identified as a significant predictor of suicide attempts [7].

The coexistence of anxiety and depression represents a significant risk factor in adolescents, increasing suicide propensity through manifestations such as sleep disturbances, palpitations, fatigue, and feelings of worthlessness [34]. Sleep duration also plays a crucial role, as both short and prolonged periods are associated with a higher likelihood of suicidal ideation and attempts, with anxiety and depression as key mediators [19]. Additionally, increased sedentary time has been linked to a greater risk of suicidal ideation and planning in adolescents [24]. It has also been evidenced that individuals with autism and high cognitive abilities exhibit a greater propensity for suicidal thoughts [8]. Other contextual factors, such as cyber victimization and perceived stress, have also been explored, with stress emerging as a mediator in suicide risk [30]. A paradigmatic case, the "Iveco" case in Spain, established a connection between digital image-based abuse and the victim’s suicide, generating significant social impact [15].

#### 4.1.2. Family and Contextual Factors

The family environment also plays a fundamental role in adolescent mental health. Economic conditions have been reported to significantly influence educational outcomes and childhood mental well-being [39]. Additionally, demographic variables such as gender, place of residence, substance use, and family context have been identified as determinants in adolescent suicidal ideation [11]. During the COVID-19 pandemic, factors such as residence in urban areas and family job loss were highlighted as critical elements in youth suicide attempts [35]. In this context, it has been evidenced that planning and attempts in individuals with suicidal ideation are influenced by sociodemographic variables, mental disorders, adverse childhood events, and previous suicidal behaviors [21]. Furthermore, emotional neglect within the family has been identified as a significant contributing factor to suicidal tendencies [7].

The relationship between child abuse, depression, and suicidal ideation has been extensively documented, with implicit self-esteem identified as a potential mediator in this relationship [31]. Similarly, the perception of gender equality and childhood trauma experiences in adolescents with depression have been analyzed, revealing that a traditional view of gender roles in mothers and adolescents is associated with more severe suicide attempts [18].

#### 4.1.3. School Factors

The school environment also plays a crucial role in adolescent mental health. Disconnection from school and bullying have been identified as significant risk factors for suicidal ideation and behaviors [26]. Additionally, elements such as victimization by physical aggression, participation in fights, and school bullying have been identified as relevant risk factors [25]. The relationship between bullying victimization, self-harm, and suicide has been analyzed, revealing gender differences in predisposition to depression, anxiety, and suicidal ideation, with depression acting as a key mediator [16].

Finally, the impact of the 13 Reasons Why series on adolescent suicide in the U.S. has been studied, identifying seasonal patterns and school-related factors as key elements in youth suicide risk. An increase in suicides was observed between March and April, coinciding with the school calendar, with no evidence of a direct effect of the program on male suicides in 2017 when seasonality and other factors were considered [33].

### 4.2. Prevention Efforts in Adolescent Suicide: A Global Perspective

Efforts to prevent adolescent suicide have been widely addressed in the international scientific literature (for a brief overview, see Appendix A, Table A2).

Research highlights the fundamental role of teacher training in tackling this issue, especially in the case of Indigenous children in Canada, where suicide has a particularly significant impact [27]. In South Korea, suicide remains the leading cause of death among adolescents; however, only 20% of students who died by suicide had received guidance or support within the school environment [20].

In contrast, school-based interventions and support mechanisms serve as protective factors, as students report that structured school activities positively influence their mental health [26]. In this regard, large-scale educational support programs have been proposed to improve adolescent well-being [35]. Likewise, the integration of Social–Emotional Learning (SEL) has been emphasized as a key component in suicide prevention programs implemented in schools across the United States [29]. These programs include teaching materials, educator guides, and assessment tools designed to incorporate emotional development into the school environment systematically.

In educational contexts such as those in North America and Europe, the presence of school counseling services and school-based mental health clinics constitutes a key component for the early identification and direct intervention of suicidal behavior in adolescents. These resources complement the preventive and educational functions of teaching staff, reinforcing a multidisciplinary approach to suicide prevention.

These findings reinforce the need to view schools as one of the most stable, accessible, and structured spaces in the lives of children and adolescents, making them a privileged setting for the implementation of suicide prevention strategies. Such intervention is not only desirable but also ethically and socially imperative. The inclusion of social-emotional education in the school curriculum can be implemented in a cross-curricular, explicit, or combined manner, depending on the priorities of the educational system, teacher training, and available resources. A common approach involves incorporating these contents into specific subjects such as Tutorial, Values Education, Citizenship, or Coexistence, where topics such as emotional regulation, conflict resolution, self-esteem, and bullying prevention can be addressed directly. Another frequent approach is the cross-curricular integration of these competencies into different academic areas through activities that promote emotional reflection, cooperative learning, or the development of empathy, such as debates, the analysis of ethical dilemmas, or collaborative interdisciplinary projects.

From a global health perspective, adolescent suicide is recognized as a serious public health issue [25]. Protective factors such as self-assessment and positive self-awareness have been shown to mitigate suicidal ideation [40]. Similarly, strengthening interpersonal relationships and addressing internalized feelings of guilt are fundamental strategies to reduce suicide risk in vulnerable adolescents [7]. In this context, promoting a school culture that values listening, respect, and mutual care is a structural component of any effective prevention policy. Practices such as school coexistence plans, active student participation in school life, and the promotion of affective bonds within the educational community help reduce loneliness and increase the perception of support—key elements in suicide prevention.

The interpersonal theory of adolescent suicide has also been the subject of analysis, with adaptations proposed to incorporate developmental processes into preventive strategies [9]. This approach reinforces the need to train teachers to identify early warning signs, provide emotional support, and activate clear, accessible protocols shared across the entire educational community. The goal is not for teachers to take on the role of mental health professionals but to empower them as the first link in an adequate protection chain.

Risk assessment tools also play a crucial role in this process. Instruments such as the PHQ-9M and the PSC-17P have been widely used to evaluate suicide risk in adolescent populations [12]. Additionally, the Columbia Suicide Severity Rating Scale has been used to assess the severity of suicidal behavior in adolescents diagnosed with depression. Alongside these, other tools such as the Perceived Gender Scale (PGS), the Childhood Trauma Questionnaire (CTQ), and the Beck Depression Inventory (BDI) have helped analyze associated psychological variables [18], which is essential for developing interventions tailored to the specific needs of each context.

From a community perspective, it has been noted that cross-sector collaboration remains insufficient, as Child Death Review (CDR) teams and schools show low levels of coordination when addressing both suicidal and non-suicidal deaths [17]. Strengthening these collaborative ties between schools, families, and the healthcare system is essential for designing more effective prevention strategies and ensuring comprehensive support for at-risk adolescents.

### 4.3. Family Experiences of Suicidal Behavior

The impact of suicide on the family environment constitutes a critical dimension in medical and psychological care. In the case of adolescents who have been hospitalized for suicidal ideation or behaviors, their return to school represents a complex process involving multiple challenges for both the adolescents and their caregivers. The main difficulties identified include academic problems resulting from school interruption, disruptions in communication dynamics with teachers and peers, and managing interpersonal relationships in a context marked by stigma and emotional vulnerability [36]. These factors can affect the adolescent’s recovery and have a significant impact on the mental health of caregivers, who must play an active role in the reintegration of the adolescent into the educational and social environment.

On the other hand, the loss of a child to suicide represents one of the most devastating experiences for a family, especially for mothers, who often assume a central role in the grieving process. The adaptation process following this type of loss is influenced by previous life events, access to support networks, and the possibility of reframing the experience within a framework of personal transformation. In this context, understanding the emotional repercussions of grief and the factors that facilitate or hinder the reconstruction of psychological well-being is essential for designing effective, evidence-based intervention strategies [37].

## 5. Discussion

Suicide among children and adolescents remains a significant public health challenge, influenced by a complex interplay of psychological, familial, educational, and contextual factors [10,32]. This systematic review highlights that depression, anxiety, suicidal ideation, and emotional disorders are strongly associated with adverse childhood experiences, particularly bullying and social isolation [12,13,21]. Furthermore, emotional resilience has emerged as a key protective factor against suicide risk, emphasizing the need for targeted interventions that promote psychological well-being and adaptive coping strategies [40].

The reviewed studies highlight the fundamental role of the family environment in adolescent mental health. Risk factors such as lack of parental care, family breakdown, and absence of emotional support significantly contribute to suicide vulnerability [7,31]. In this regard, preventive strategies should incorporate family- and community-centered intervention programs aimed at early detection and psychosocial support for at-risk youth. Additionally, socioeconomic disadvantage has been identified as a determinant of suicidal ideation, necessitating specific interventions that address the unique needs of vulnerable communities [35,39]. However, the predominance of studies in countries where most research has been conducted, such as the United States, China, and Spain, reveals an imbalance in global research efforts. This underscores the need to expand investigations into regions with limited mental health resources, ensuring a more comprehensive understanding of suicide risk factors across diverse sociocultural contexts.

The school environment plays a fundamental role in preventing adolescent suicide. Exposure to bullying, social exclusion, and media representations of suicide exacerbate vulnerability [16,26,33]. Implementing Social and Emotional Learning (SEL) programs in educational institutions represents a promising strategy for enhancing emotional intelligence and reducing suicidal behavior among adolescents [29]. Additionally, evaluating the long-term efficacy of prevention strategies is essential to ensure their sustainability and adaptability across different educational and social contexts.

From a preventive perspective, adopting a multidisciplinary approach is essential. This includes integrating advanced diagnostic tools, such as scientifically validated suicide risk assessment scales [12,18], and developing personalized interventions that promote positive self-awareness and strengthen support networks [7,40]. Public health policies should prioritize early detection mechanisms alongside improving family and school support systems to mitigate suicide risk among adolescents. Effective collaboration between mental health professionals, educators, and families is crucial for addressing this issue comprehensively and sustainably over time.

Research indicates that organized school adaptation after a crisis is fundamental for preventing relapses and facilitating the social reintegration of adolescents [36,37]. To address this public health issue effectively, post-crisis support programs and efficient early identification mechanisms must be strengthened. However, future studies should focus on evaluating the long-term impact of these interventions, particularly regarding their adaptation to diverse sociocultural and economic contexts.

Finally, it is important to highlight several recent studies that strongly support the contribution of research conducted in Latin America and Africa to the understanding of adolescent suicide [41,42,43,44]. Some identify risk and protective factors in Latin American youth based on systematic reviews of scientific studies [41]. Others systematize interventions implemented with adolescents in various Latin American and European countries, providing a practical comparative framework for transferring good practices [42]. Data from several West African countries have also been analyzed using international school-based surveys [43], and suicidal behaviors have been explored among adolescents in specific contexts of sub-Saharan Africa [44]. All of these studies emphasize the role of school, family, and social factors in suicide risk, aligning with the patterns described in this review and reinforcing their validity from other geographical realities.

## 6. Conclusions

The results of this systematic review highlight the magnitude and complexity of suicide in adolescence, emphasizing its multifactorial nature and the constant interaction between psychological, family, school, and social variables. This study shows that emotional disturbances such as depression, anxiety, or mood disorders, far from occurring in isolation, are intensified by previous experiences of trauma, situations of emotional neglect, and school contexts marked by bullying and disengagement. These factors significantly increase the vulnerability of adolescents, especially when they lack personal and social coping resources. In this scenario, the school appears as a key environment because of its capacity for early detection and its preventive potential if emotional education programs and teacher training in mental health are incorporated. The role of the teacher should not be limited to identifying warning signs but also to building safe, welcoming, and caring educational environments.

At the same time, the family continues to be a determining factor. Communication dynamics, the presence or absence of emotional support, and the ability of families to provide emotional support to adolescents are decisive in the prevention and recovery processes after a crisis. For this reason, it is necessary to articulate intervention strategies that integrate the community dimension, favoring collaboration between health professionals, schools, and families. Likewise, gaps in institutional coordination are identified that limit the effectiveness of responses to suicidal behavior, especially regarding school reintegration after hospitalization or the accompaniment of grief in cases of completed suicides. These situations require specific protocols that are sensitive to the context and not only respond to clinical urgency but also accompany the processes of emotional adaptation from a comprehensive and respectful perspective.

At the same time, there is a clear geographical imbalance in scientific production that urgently needs to be addressed. Most studies are concentrated in countries with consolidated mental health systems, which makes the realities of many regions invisible. Resources are scarce, and prevention strategies are still in their infancy. Broadening the research focus to diverse contexts is a priority in order to develop truly inclusive and effective responses.

## Figures and Tables

**Table 1:** Search Equations.

Database	Search Equation	Location	N
Scopus	(“suicide”) AND (“child*” OR “early child*”) AND (“educat*”)	Title Abstract KW	2620
WoS	(“suicide”) AND (“child*” OR “early child*”) AND (“educat*”)	Abstract	1221
Dialnet Plus	(suicid*) AND (infancia OR niñ* OR “primera infancia”) AND (educac*)	*	63
Total			3904

Note. The term “childhood” was included as a key concept during the literature search to ensure a comprehensive review encompassing studies with samples ranging from childhood to early adolescence. This decision was based on evidence indicating that many risk factors associated with adolescent suicide originate in earlier developmental stages. * Not applicable.

**Table 2:** Characteristics of the documents.

Authors	Year	Country	Method*	Sample Size	Study Design
Austin et al. [7]	2022	United States Canada	QUAN	*N* = 372 participants.	Cross-sectional
Casten et al. [8]	2023	United States	QUAN	Specialized clinical sample: • N = 1074 assessments of children with high cognitive abilities (IQ ≥ 120) in a specialized clinic (2009–2019). SPARK (Simons Foundation Powering Autism Research for Knowledge) + ABCD (Adolescent Brain Cognitive Development Study) combined sample: • *N* = 16,049 participants (children with and without autism). • Within SPARK: *N* = 1983 (for genetic analysis). • Analysis with parents: *N* = 736.	Cross-sectional
Chung et al. [9]	2022	United States	QUAN	*N* = 9421 participants from the Add Health National Longitudinal Study of Adolescent to Adult Health.	Longitudinal
Davis et al. [10]	2022	United States	QUAN	*N* = 82,531 participants (aged 12–17 years).	Cross-sectional
Ghosh and Bhattacharjee [11]	2022	India	QUAN	*N* = 500 participants (aged 16–18 years).	Cross-sectional
Holcomb et al. [12]	2022	United States	QUAN	*N* = 5411 participants (aged 11–17 years).	Cross-sectional
Husky et al. [13]	2022	Europe	QUAN	*N* = 5183 participants (aged 6–11 years).	Cross-sectional
Hyland et al. [14]	2022	United States	QUAN	*N* = 507 participants (aged 7–18 years).	Cross-sectional
Idoiaga et al. [15]	2022	Spain	QUAN	1895 tweets that contained the word IVECO and were published in Spain.	Qualitative cross-sectional analysis of social media content
Islam et al. [16]	2022	Australia	QUAN	*N* = 2522 participants (aged 12–17 years).	Cross-sectional
Joshi et al. [17]	2023	United States	QUAN	*N* = 32,591 reviewed deaths of children and adolescents (aged 5–20 years) occurring between 2005 and 2017. The sample was divided into three age groups according to school level: • Primary education: *n* = 7004 cases; • Secondary education: *n* = 6932 cases; • Secondary education: *n* = 18,655 cases. Among these deaths, *n* = 6208 (19.0%) were due to suicide.	Cross-sectional
Kafali et al. [18]	2022	Turkey	QUAN	*N* = 84 adolescent girls.	Cross-sectional
Kim et al. [19]	2023	Korea	QUAN	*N* = 544 participants (analyses data on students who died by suicide in South Korea between 2016 and 2020).	Cross-sectional
Kim et al. [20]	2022	Korea	QUAN	*N* = 54,948 participants (aged 12–18 years).	Cross-sectional
Koh et al. [21]	2023	Singapore	QUAN	The total sample size for SMHS 2016 was *N* = 6126, with a response rate of 69.0%. The prevalence of suicide planning was 17.7% (*n* = 84), with 80.4% of these cases occurring within a year of suicidal ideation. The prevalence of suicide attempts was 10.6% (*n* = 47), with 83.9% of cases occurring within a year of suicidal ideation.	Cross-sectional
Lawson et al. [22]	2022	Mexico	QUAN	*N* = 674 participants (aged 12–21 years).	Longitudinal
Liu et al. [23]	2023	China	QUAN	*N* = 1913 participants (aged 11–19 years).	Cross-sectional
Ma et al. [24]	2022	United States	QUAN	*N*= 146,345 participants from 54 countries (aged 12–15 years).	Cross-sectional
Mahumud et al. [25]	2022	77 countries	QUAN	*N*= 251,763 participants (aged 11–17 years) across 77 countries.	Cross-sectional
Marraccini et al. [26]	2022	United States	QUAL	*N* = 64 participants, distributed as follows: • Adolescents previously hospitalized for suicidal crisis: *n* = 19. • Parents of these adolescents: *n* = 19. • School professionals (counselors, psychologists, etc.): *n* = 19. • Hospital professionals: *n* = 7.	Cross-sectional
McVittie and Ansloos [27]	2023	Canada	QUAL	*N* = 7 participants (female educators aged 40–60 years).	Cross-sectional
Okada et al. [28]	2022	Japan	QUAN	*N* = 5641 participants (aged 7–18 years).	Cross-sectional
Posamentier et al. [29]	2023	United States	Literature analysis.		
Quintana-Orts et al. [30]	2022	Spain	QUAN	*N* = 1821 participants (aged 12–17 years).	Cross-sectional
Reid-Russell et al. [31]	2022	United States	QUAN	*N* = 240 participants (aged 8–16 years).	Longitudinal
Rihmer et al. [32]	2022	Hungary	Literature analysis.		
Romer [33]	2023	United States	QUAN	The study did not use a direct individual sample but analyses aggregated population-based data from suicide mortality records. The sample was composed of the total number of suicides recorded weekly between 2013 and 2018 for the following groups: • Adolescents aged 10–19 years; • Young people aged 20–29 years (with a focus on men aged 20 to 24).	Longitudinal
Shen and Wang [34]	2023	China	QUAN	*N* = 9300 participants.	Cross-sectional
Valdez-Santiago et al. [35]	2022	Mexico	QUAN	*N* = 15,460 participants. Among them: • *n* = 3547 adolescents. • *n* = 11,913 adults.	Cross-sectional
Vanderburg et al. [36]	2023	United States	QUAL	*N* = 19 participants (in-depth interviews with caregivers, after hospitalization of an adolescent).	Cross-sectional
Whalen and Tisdell [37]	2023	United States	QUAL	*N* = 4 participants (mothers who lost a child to suicide).	Cross-sectional
Wong et al. [38]	2022	Hong Kong	QUAN	*N* = 35 recorded cases of student suicide (aged 10–20 years).	Cross-sectional
Xiao et al. [39]	2023	China	QUAN	*N* = 2531 participants (aged 8–16 years).	Cross-sectional
Zou et al. [40]	2022	China	QUAN	*N* = 10,183 participants (aged 11–18 years).	Cross-sectional

Note. Method*: QUAN: Quantitative methodology; QUAL: Qualitative methodology.

## Data Availability

The data supporting this study’s findings are available from the corresponding author upon reasonable request.

## Appendix A

**Table A1:** Summary of the risk factors for suicidal behavior collected in the studies.

Category	Variables/Components	Main Results	References
**Psychological and mental health factors**	- Depression, anxiety, and feelings of loneliness. - Mood disorders. - Dimensions of the HiTOP model (fear, distress, externalization, thought disorders, and traumatic stress).	High levels of depressive and anxious symptoms, along with traumatic experiences, are associated with increased suicidal ideation and behaviors.	Holcomb et al. (2022) [12]; Koh et al. (2023) [21]; Hyland et al. (2022) [14]; Mahumud et al. (2022) [25]; Wong et al. (2022) [38]; Okada et al. (2022) [28].
**Personality traits and temperament**	- Insensitivity; - Negative affect; - High emotionality; - Effortful control (protective factor).	Traits of high emotionality and negative affect are linked to greater risk, while greater effortful control acts as a protective factor.	Lawson et al. (2020) [22]; Liu et al. (2023) [23].
**Behavior and mediating factors**	- Sleep quality and duration; - Sedentary behavior; - Interpersonal microaggressions.	Altered sleep duration (both short and long) and increased sedentary behavior, along with experiences of microaggressions, mediate the relationship between symptoms and risk.	Kim et al. (2023) [19]; Ma et al. (2022) [24].
**Family and contextual factors**	- Socioeconomic determinants; - Emotional neglect and child abuse; - Parental separation; - Traditional perceptions of gender roles.	Economic environment and early experiences (neglect, abuse, separation) significantly influence vulnerability to suicidal ideation and planning.	Xiao et al. (2023) [39]; Valdez-Santiago et al. (2023) [35]; Reid-Russell et al. (2022) [31]; Kafali et al. (2022) [18].
**School Factors**	- Bullying and school harassment; - Physical aggressions; - Lack of support and connection with peers; - Influence of media content.	Negative experiences in the school environment, both due to bullying and aggression as well as exposure to particular media content, increase the risk of suicide.	Marraccini et al. (2022) [26]; Islam et al. (2022) [16]; Romer (2023) [33].

**Table A2:** Summary of suicide prevention strategies and interventions collected in the studies.

Strategy/Intervention	Description	Tools/References	Expected Results
**Interventions in the school setting**	Educator training and Social–Emotional Learning (SEL) programs to improve climate and early detection in the school environment.	SEL programs; school support initiatives. Marraccini et al. (2022) [26]; Posamentier et al. (2023) [29].	Early detection of risk, reduction in bullying, and improvement of the school environment.
**Diagnostic tools**	Use of validated instruments to assess and monitor suicidal risk in adolescents.	PHQ-9M, PSC-17P, and Columbia Suicide Severity Scale. Holcomb et al. (2022) [12]; Kafali et al. (2022) [18].	Accurate diagnosis and monitoring of risk, facilitating timely interventions.
**Person-centered interventions**	Encouragement of positive self-evaluation, strengthening of interpersonal relationships, and reduction in self-stigma.	Individual and group therapies; psychosocial interventions. Zou et al. (2022) [40]; Austin et al. (2022) [7].	Reduction in suicidal ideation and strengthening of individual resilience.
**Accompany adaptation and mourning processes**	Support during post-crisis school reintegration and in the management of family grief, facilitating adaptation and communication after suicidal episodes.	Family therapeutic interventions; psychological support programs. Vanderburg et al. (2023) [36]; Whalen and Tisdell (2023) [37].	Improved post-crisis adaptation and mitigation of traumatic effects in the family environment.
